# Changes in Body Weight and Serum Albumin Levels in Patients Requiring Home Long-term Oxygen Therapy

**DOI:** 10.31372/20200504.1113

**Published:** 2021

**Authors:** Naomi Kayauchi, Eiji Ojima, Katsunori Kagohashi, Hiroaki Satoh

**Affiliations:** University of Tsukuba, Mito Medical Center, Ibaraki, Japan

**Keywords:** nutrition, albumin, body weight, home long-term oxygen therapy, chronic respiratory failure, chronic heart failure, chronic obstructive pulmonary disease, interstitial pneumonia, nursing

## Abstract

**Purpose:** To investigate the long-term changes in body weight and serum albumin levels in patients with respiratory failure, and those with chronic heart failure, who were treated with home long-term oxygen therapy (LTOT) to understand the current status and contribute to future measures.

**Methods:** Patients with chronic obstructive pulmonary disease (COPD), those with interstitial pneumonia (IP), and those with chronic heart failure (CHF) undergoing home LTOT for 6 months or more between January 2011 and January 2019 were included in the study. Body weight and serum albumin levels were assessed at the start of home LTOT and at the end of the observation period, a minimum of 6 months after commencing home LTOT.

**Results:** Sixty-two patients (29 COPDs, 23 IPs, and 10 CHFs) were included. In COPD patients and IP patients, body weight decreased (*P* = 0.0017, *P* = 0.0018, respectively, Wilcoxon signed-rank test). Serum albumin levels decreased in IP patients (*P* = 0.0185) but not in COPD patients. There was neither significant decrease in body weight nor serum albumin levels in patients with CHF.

**Conclusion:** Chronic respiratory failure patients who have home LTOT were likely to have a decreased nutritional status. In order to provide prolonged home LTOT, medical staff need to pay close attention to the nutritional status of patients receiving home LTOT.

## Introduction

Home long-term oxygen therapy (LTOT) is an inhaling oxygen therapy for patients with chronic respiratory failure (CRF) or chronic heart failure (CHF) whose arterial blood oxygen levels have fallen below a certain level (Jacobs et al., 2018; Medical Research Council Working Party, 1981; Nocturnal Oxygen Trial Group, 1980). In Japan, home LTOT is performed in patients with PaO_2_ of 55 mmHg or less, and those with PaO_2_ of 60 mmHg or less who exhibit marked hypoxemia during exercise (Japanese Respiratory Society Lung Physiology Expert Committee, 2008). Home LTOT is also given to patients with CHF of New York Heart Association (NYHA) grade 3 or higher (Japanese Circulation Society, 2017). It is well known that patients with CRF and those with CHF have weight loss and malnutrition (Cavaillès et al., 2013; Yang, Li, & Gao, 2020). The causes are thought to be increased energy requirements due to CRF and CHF and decreased energy intake due to appetite loss. In particular, many studies on nutritional status in patients with chronic obstructive pulmonary disease (COPD) have been reported (Cavaillès et al., 2013). If patients with oxygen therapy were in a hospital or nursing facility, they might be able to secure a nutritionally controlled diet, but there was a question that it might be difficult to provide adequate nutrition at home. Also, because of the patient’s pickiness of food, there was a question as to whether they could properly accept diets at home.

Many previous studies used serum albumin level as nutritional indicators (Cano et al., 2002; Tóth, Tkácová, Matula, & Stubna, 2004). However, some published evidence shows that low serum albumin level alone should not be used as indicators for malnutrition as it is affected more significantly by other conditions such as production disorders, increased catabolism, and extracorporeal leakage (Akner & Cederholm, 2001; Don & Kaysen, 2004; Onishi, 2007). Malnutrition or protein–energy malnutrition (PEM) is defined as a range of pathological conditions arising from coincident lack of dietary protein and/or energy in varying proportions (Haider & Haider, 1984). Evaluation of serum albumin alone overlooks Marasmus-type PEM, one of the two types of PME, and in order to eliminate overlook of this type of nutritional disorder, it is necessary to evaluate in combination with anthropometric indicators (Haider & Haider, 1984). In this study, therefore, in addition to serum albumin, we evaluated body weight, which is the most basic anthropometric indicator.

There were articles that studied nutritional indicators before the start of LTOT and subsequent respiratory function (Cano et al., 2002) and prognosis (Tóth et al., 2004). They used nutritional indicators within 2 years of the start of LTOT, and they did not compare nutritional indicators over time (Cano et al., 2002; Tóth et al., 2004). To the best of our knowledge, therefore, there was no report examining long-term changes in nutritional status in patients receiving home LTOT. To investigate the actual nutritional status of patients with good respiratory control and long-term survival, in this study, we included all patients who were followed up as long as possible.

The most common patients receiving home LTOT have been COPD patients, however, many patients with interstitial pneumonia (IP) and those with CHF also receive this treatment. Therefore, this study investigated body weight and serum albumin levels in patients with LTOT due to these three diseases. Considering the results of many previous studies (Cano et al., 2002; Suzuki et al., 2018; Tóth et al., 2004; Zhou et al., 2013), we speculated that CRF patients due to COPD would display the most prominent decrease in nutrition. With the aim of understanding the current status and contributing to future measures, we investigated the long-term changes in body weight and serum albumin levels in patients requiring home LTOT.

## Methods

This exploratory study had a retrospective, nonexperimental observation design. We investigated the medical records of all the patients with CRF and those with CHF who required home LTOT for more than 6 months in a tertiary hospital in Japan. The definition of respiratory failure was PaO_2_ of 55 mmHg or less, and PaO_2_ of 60 mmHg or less who exhibit marked hypoxemia during exercise. Many respiratory diseases cause CRF (Japanese Respiratory Society Lung Physiology Expert Committee, 2008). The term “CRF” used in this study was limited to CRF caused by COPD and to CRF caused by IP. Malignant diseases have effects on serum albumin levels and body weight. Therefore, patients with malignant disease were not included in this study to rule out the effects of malignant disease on nutritional status. Patients receiving home oxygen therapy for a short period of time (6 months or less) were not included in the study because this study was aimed at investigating the status of patients with “long-term” oxygen therapy. COPD was diagnosed according to the American Thoracic Society diagnostic criteria (American Thoracic Society, 1987). In this study, IP included idiopathic pulmonary fibrosis (Travis et al., 2013) as well as IP associated with collagen diseases. CHF was diagnosed according to NYHA criteria. We investigated medical records in CHF patients with NYHA grade 3 or higher. We investigated all patients who were treated with home LTOT between January 2011 and January 2020 in our hospital. Changes in body weight and serum albumin levels were assessed at the start of home LTOT and at the end of the observation period.

Body weight and serum albumin levels at the time of start of LTOT and at the time of final visit were noted from medical chart. The patients visited the hospital once a month after the initiation of LTOT. The body weight of patients was measured by nurses at the time of initiation of LTOT and at the time of each hospital visit. Serum albumin was determined using conventional methods.

Differences in proportions between two independent groups were compared by the *χ*^2^ test. The Kruskal–Wallis test was used to compare three groups of sample data. The Wilcoxon signed-rank test was used for analysis of index changes in the same patient. *P* < 0.05 was considered to indicate a statistically significant difference.

This retrospective noninterventional study conformed to the Declaration of Helsinki and the Ethical Guidelines for Clinical Studies issued by the Ministry of Health, Labor and Welfare in Japan. Written comprehensive consent for a noninterventional retrospective study was obtained from each patient. Analysis of the medical records of patients who received LTOT was approved by the ethics committee in our institute.

## Results

### Patient Characteristics

**Figure 1:**
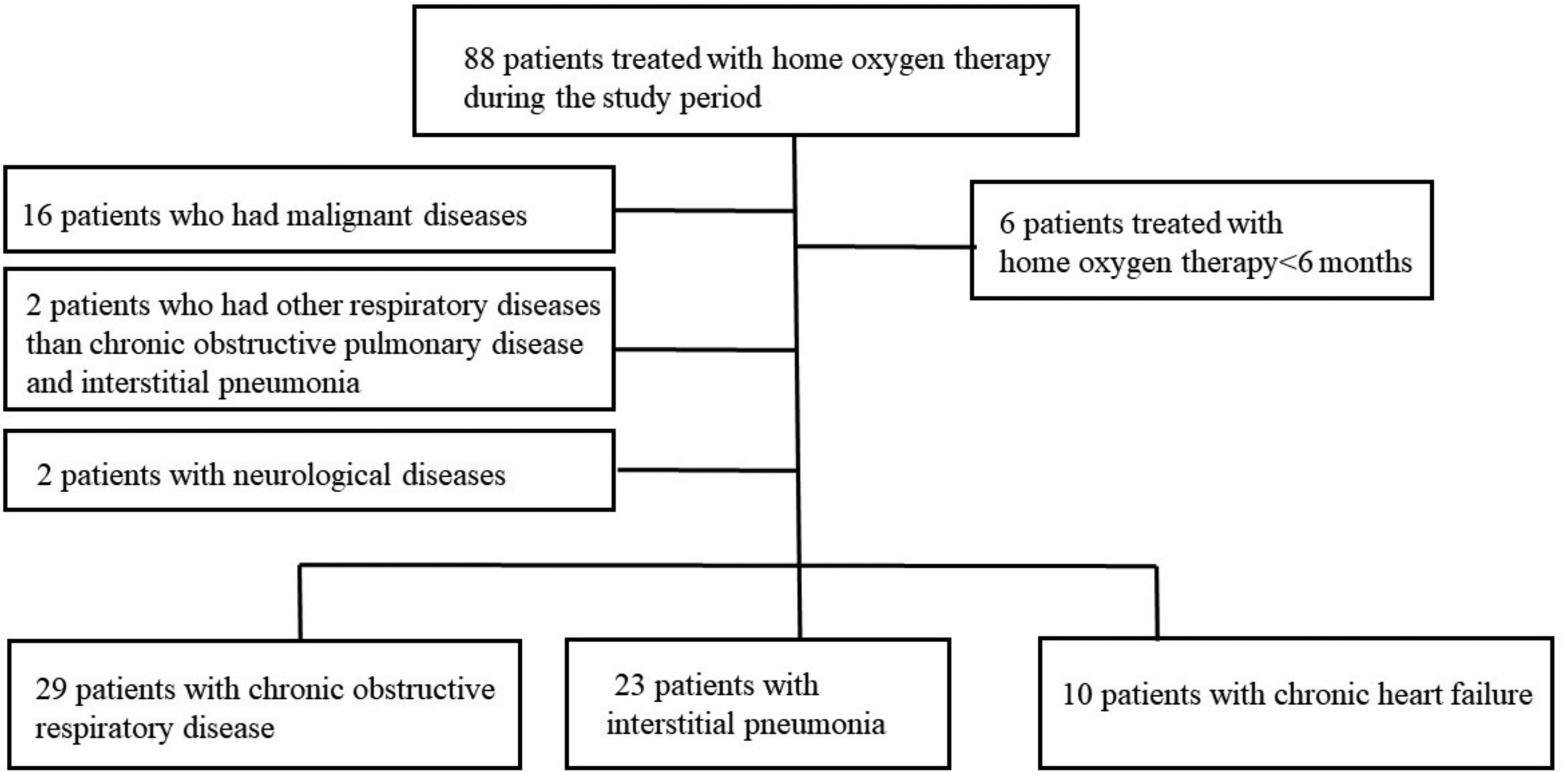
Study participant flow chart.

During the study period, 88 patients had home LTOT. Among them, 16 patients with malignant disease, two patients who had other respiratory diseases than COPD and IP, two patients with neurological diseases, and six patients treated with home LTOT < 6 months were excluded from this study (Figure 1). Table 1 shows the characteristics of the 29 patients with COPD, 23 patients with IP, and 10 patients with CHF included in the study. There were no significant differences in characteristics among these three groups of patients. In our country, normal ranges of serum albumin level are 3.7–5.0 g/dL, and the average weight (standard deviation) for men aged 70 and over was 62.7 (9.3) kg for men and 51.3 (8.8) kg for women (Ministry of Health, Labor and Welfare of Japan, 2018).

**Table 1:** Characteristics of Patients with COPD, IP, and CHF

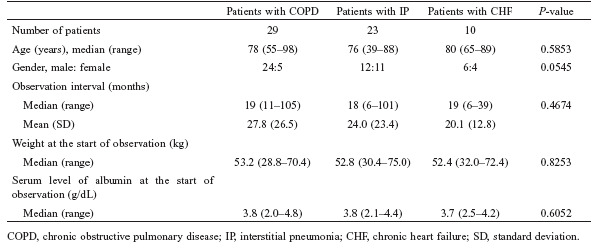

### Body Weight and Serum Albumin Level Changes in each Patient

**Figure 2:**
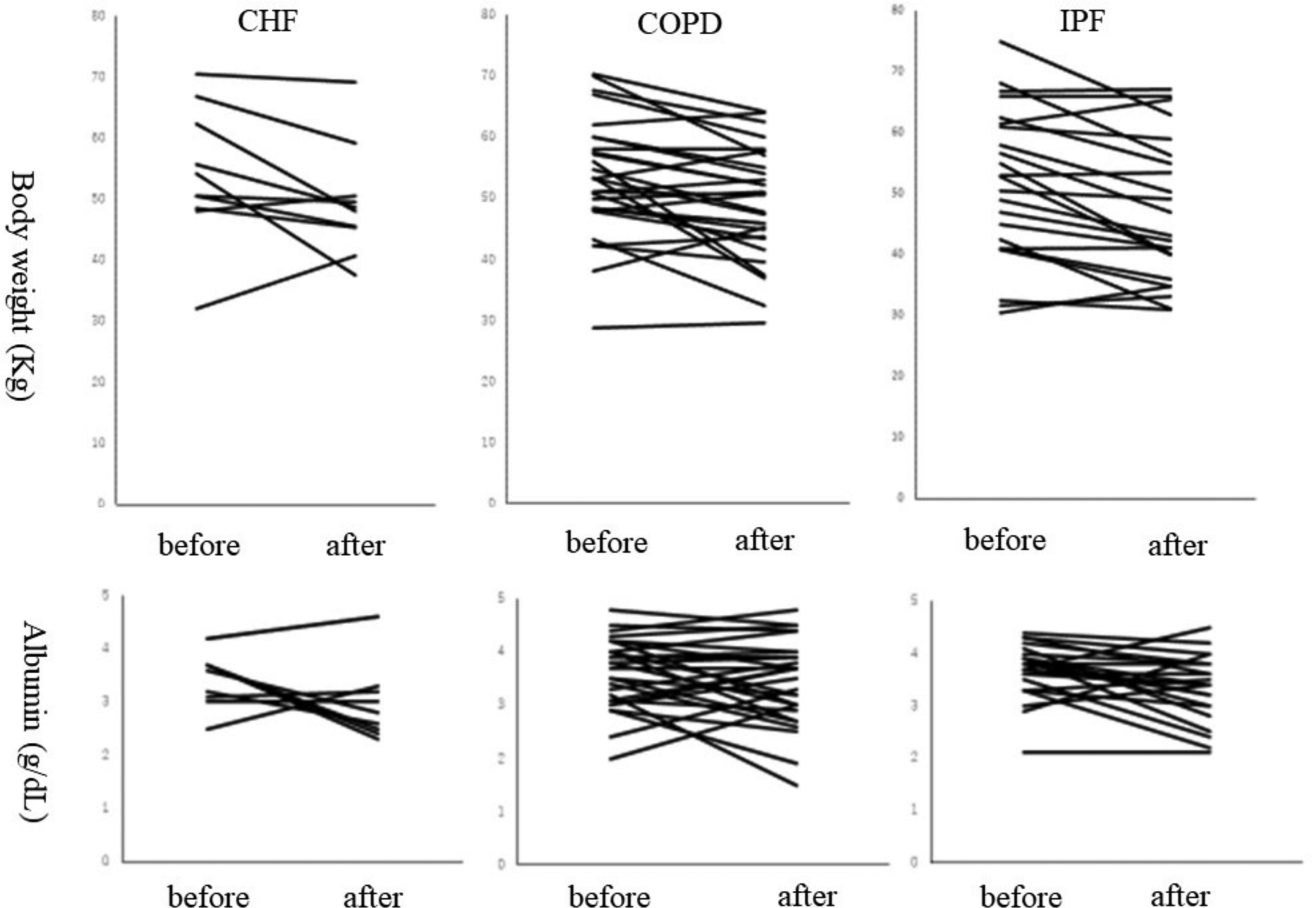
Comparison of body weight and serum albumin level at the start of home LTOT (left) and at the end of the observation period (right) in patients with COPD, those with IP, and those with CHF.

Focusing on each patient, the changes in body weight and serum albumin levels before and the end of the observation period of LTOT are shown in Table 2 and Figure 2. In COPD patients and those with IP, body weight decreased over the observation period (*P* = 0.0017, *P* = 0.0018, respectively; Wilcoxon signed-rank test). However, the body weight in patients with CHF did not change. Serum albumin levels decreased in patients with IP over the observation period (*P* = 0.0185; Wilcoxon signed-rank test), but serum albumin levels in patients with COPD and those with CHF did not change.

**Table 2:** Comparisons of Body Weight and Serum Levels of Albumin at the Start of Home LTOT and at the End of the Observation Period in Patients with COPD, IP, and CHF

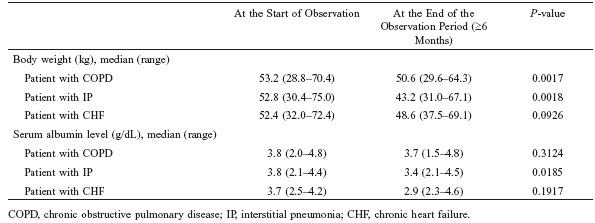

### Comparison of the Changes across the Three Groups (COPD, IP, and CHF)

Focusing on the differences in patient groups, the changes in body weight and serum albumin levels before and at the end of the observation period of LTOT are shown in Table 3 and Figure 2. The extent of changes in body weight from the start of home LTOT to the end of the observation period among these patient groups were not significantly different (*P* = 0.7371; Kruskal–Wallis test). There was no difference in the extent of change in serum albumin levels among the groups (*P* = 0.4925; Kruskal–Wallis test).

**Table 3:** Comparison of the Extent of Changes in Body Weight and Serum Albumin Levels from the Start of Home LTOT to the End of the Observation Period in Patients with COPD, IP, and CHF

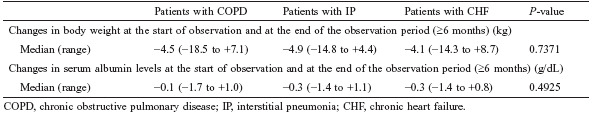

## Discussion

In recent years, the concepts of cachexia, sarcopenia, and frailty have become common, and studies based on these concepts have been conducted in the area of cancer treatment (Jain, Coss, Whooley, & Phelps, 2020; Kidd, Skrzypski, Jamal-Hanjani, & Blyth, 2019; Kurishima, Watanabe, Ishikawa, Satoh, & Hizawa, 2017; Nakamura et al., 2018; Phillips, Hug, Allan, & Ezhil, 2019). We have also been interested in cachexia and sarcopenia in lung cancer patients (Kurishima et al., 2017; Nakamura et al., 2018; Sasatani, Okauchi, Ohara, Kagohashi, & Satoh, 2020). Clinical trials of anticachexia drugs were conducted recently, and these are expected to be available for clinical use in the near future (Hamauchi et al., 2019). Attention should be paid, not only to cancer cachexia, but also to cachexia associated with CRF and CHF. Physiologically, one of the major causes of poor nutritional status in patients with CRF and those with CHF is the increased nutritional demand required to maintain homeostasis under inefficient respiratory and circulatory conditions (Cavaillès et al., 2013; Yang et al., 2020). Loss of appetite due to diminished digestive function due to chronic hypoxia, reduced activity due to decreased exercise tolerance, and loss of muscle mass are also associated with weight loss and decreased serum albumin levels (Cavaillès et al., 2013). Regarding nutritional status, many studies have been reported on COPD patients, and body weight, body mass index, and serum albumin levels have been used as indices for evaluating nutrition in these patients (Cano et al., 2002; Suzuki et al., 2018; Tóth et al., 2004; Zhou et al., 2013). Among them, there were only two articles on pretreatment nutritional status and prognosis of patients undergoing LTOT (Cano et al., 2002; Tóth et al., 2004). Cano et al. estimated the prevalence of malnutrition in outpatients on LTOT or home mechanical ventilation (Cano et al., 2002). In their study, body weight and serum albumin were noted at the time of enrolment and 1 year prior to the enrolment. They concluded that malnutrition was highly prevalent in home-assisted respiratory patients (Cano et al., 2002). In the article by Tóth et al., prognostic value of pre-LTOT, low body mass index, and low albumin were related to worse 2-year survival (Tóth et al., 2004). However, to the best of our knowledge, there was no report examining changes in nutritional status and serum albumin levels in patients receiving home LTOT.

In the present study, we obtained the following four results. (1) In three groups of patients investigated, the median age of patients receiving home LTOT was late seventies. In these three groups of patients, the median body weight at the start of home LTOT was 52–53 kg, and the median serum albumin level was 3.8 g/dL. These results were as predicted before the start of this research, confirming that home LTOT often involved elderly patients who were relatively thin and had a slightly reduced nutritional status. (2) Weight loss was significant in COPD patients and those with IP, but not in those with CHF. In COPD patients undergoing home LTOT, we found a weight loss of 2.6 kg (median) over the observation period of 19 months (median). IP patients had a weight loss of 9.6 kg (median) at follow-up of 18 months (median). On the other hand, CHF patients had a weight loss of 4 kg (median), which was not statistically significant. It might be due to the difference in the observation period of between CHF patients and those with COPD and IP. The lack of weight loss in patients with CHF was intriguing, but the reason could not be determined. (3) The serum albumin level was significantly decreased in IP patients. The median decrease was 0.4 g/dL. In IP patients, the effects of administration of corticosteroid on appetite and catabolism were considered. Therefore, we compared the changes in body weight and serum albumin levels between patients taking oral corticosteroids and those not taking them, but there was no difference between them (*P* = 0.9509, *P* = 0.1842, respectively). We speculated that serum albumin levels would decrease in patients with COPD; however, the median decrease was 0.1 g/dL, which was not statistically significant. The reasons for this result were unknown. However, because undernutrition in COPD patients is well known, we supposed that intervention on nutritional condition, such as the intake of oral dietary supplements, affected the results. In CHF patients, the median decrease in serum albumin level was 0.8 g/dL, which was not statistically significant. (4) We compared the changes in body weight and serum albumin level before and after the observation period among three groups of patients. There was no significant difference in the extent of decrease of these indices between the three patient groups. The above four results can be summarized into the following two points. First, it is important to take care to maintain the nutritional status of patients treated with LTOT. In addition, active treatment, including administration of anticachexia drugs, which can be prescribed in the near future, can be desired. Second, no significant decrease in body weight and serum level of albumin was observed in patients with CHF. It was interesting to note that CHF patients undergoing home LTOT might have different nutritional dynamics than those with CRF. We expect that there will be increased interest in research in this area.

Despite some interesting findings, this study had limitations. First, this was a retrospective study in a single institute, and included a small number of patients. Second, information on nutritional supplements and rehabilitation for muscle maintenance was not considered. Third, detailed information on patient comorbidities was lacking. However, this study represented the current state of medical care, and might provide useful information on the change in nutritional status of patients with CRF and CHF who were treated with home LTOT, and help plan future research in this area.

## Conclusions

CRF patients requiring home LOT are more prone to undernutrition. In order to provide prolonged home LTOT, medical staff need to pay close attention to the nutritional status of patients receiving home LTOT.

## Author Contributions

NK, KK, and HS designed the study. NK, EO, KK, and HS collected the data. NK, KK, and HS analyzed the data. NK, KK, and HS prepared the manuscript. All authors approved the final version for submission.

## Declaration of Conflicting Interests

The authors declared no potential conflicts of interest concerning the research, authorship, or publication of this article.

## Ethical Considerations

This study conformed to the Ethical Guidelines for Clinical Studies issued by the Ministry of Health, Labor and Welfare of Japan. Written comprehensive consent for a noninterventional retrospective study was obtained from each patient. Analysis of the medical records of lung cancer patients was approved by the Ethics Committee in Mito Medical Center, University of Tsukuba Hospital (NO 16–19).
